# A Semester of Hatha Yoga Has Comparable Effects to Physical Education for Children in Terms of Physical Activity and Psychosocial Indicators

**DOI:** 10.3390/children11070826

**Published:** 2024-07-06

**Authors:** Claudio R. Nigg

**Affiliations:** Department of Health Science, Institute of Sport Science, University of Bern, 3012 Bern, Switzerland; claudio.nigg@unibe.ch; Tel.: +41-31-684-4188

**Keywords:** physical activity, yoga, physical education, schoolchildren

## Abstract

Background/Objectives: As research on yoga with school children is growing, this study investigated the effects a semester of yoga versus physical education on children’s physical activity and psychosocial indicators. Methods: Physical activity and psychosocial variables were assessed at Time 1 (January/February) and Time 2 (April/May) through self-report surveys (*n* = 157; 63% female, age: m = 10.38, sd = 0.81 years) for the intervention (Hatha yoga classes) and control groups (standard physical education classes). The intervention group was also observed regarding pedometer and System for Observing Fitness Instruction Time data. Results: The results revealed a trend towards an increase for the control and a decrease for the intervention group in stress levels. The pedometer results showed a trend towards an increasing number of steps/min. Skill practice had the greatest increase with time dedicated to that activity. The observation results revealed a significant difference in activity from Time 1 to Time 2. The major differences were decreases in sitting and being very active, and an increase in walking. Conclusions: Given the observation data and our study design limitations, the study results showed similarities between Hatha yoga and physical education in terms of increasing physical activity levels and psychosocial variables; thus, yoga may be a viable alternative to children’s physical education in this regard.

## 1. Introduction

Previous studies have demonstrated that students who participate in physical activity (PA) programs experience improved psychological health, concentration, and academic achievement [1]. Yoga is a form of PA that includes foundations such as postures called asanas, breathing control techniques called pranayama, and meditation along with mindfulness practices. There are a variety of different practices of yoga, but Hatha yoga was the chosen yoga practice for this project to help develop physical health, emotional stability, and self-awareness through the unity of the foundations of yoga [2]. The yoga teacher for the students used the Yoga Ed K-8 Teacher’s Guide to gain knowledge about how to use Hatha yoga in the context of the classroom.

The effects of yoga on school children are a growing area of research; however, little is known about the value of yoga as a form of physical education for children. Hagins, Haden, and Daly [3] conducted a randomized controlled trial (RCT) investigating the effects of yoga on stress reactivity in sixth-grade students, and demonstrated no significant differences in stress reactivity for the yoga class compared to the physical education class. In another RCT, Chaya et al. [4] investigated the effects of yoga on the cognitive abilities of 7–9-year-old schoolchildren from a socioeconomically disadvantaged background and found that the yoga was as effective as PA in improving child cognitive performance. Primary education has a significant impact on human development as 10 to 15 of our early years are spent in the academic environment. Within that timeframe, we enter our formative years, which will guide us into who we will be and how we will interact with others. Having the adequate skill set for how to interact with others and how to deal with our own emotions is crucial for success and survival from both professional and personal perspectives [5].

While there is relatively limited research on youth yoga, many studies have investigated the benefits of yoga for adults. A systematic review on the effects of yoga on risk profiles and related clinical outcomes for Type 2 Diabetes mellitus (DM 2) found that, overall, the existing studies show beneficial changes and improvements to risk factors [6,7,8]. Positive changes in risk factors were observed for glucose tolerance and insulin sensitivity, lipid profiles, anthropometric characteristics, blood pressure, oxidative stress, coagulation profiles, sympathetic activation, and pulmonary function, as well as an improvement in specific clinical outcomes [6,8]. Additionally, the consistent practice of yoga has been shown to be beneficial to emotional well-being, stress management [7], depression and anxiety [9,10], and global PA levels [11].

The purpose of this study was to investigate the effects of participating in a Hatha yoga class versus a regular physical education (PE) class on levels of PA and psychosocial variables among elementary school aged children. The yoga class was hypothesized to increase PA levels and improve psychosocial variables compared to standard physical education classes.

## 2. Materials and Methods

### 2.1. Sample

All students at the participating elementary school (kindergarten to grade 6) were eligible to participate. Written parental consent and student assent were required. Grade 4–6 classes from each grade were randomly assigned to one of two groups (with 4 classes being randomly placed in the intervention group and 2 randomly placed in the control group). All grade K-3 classes were allocated to the intervention group as the comparison measurement method (survey) was not validated for this grade group. At Times 1 and 2, the intervention group was assessed using a survey [grades 4–6], pedometers [grades K-6], and System for Observing Fitness Instruction Time (SOFIT) [grades K-6], and the control group was assessed using a survey [grades 4–6] only.

A total of 157 students (63% females, age: m = 10.38 years, sd = 0.81 years) from twelve classes returned consent forms for participation and were placed in groups accordingly (see Table 1). During the spring 2013 academic year, participating students from both the intervention and control groups completed a self-report survey. For the SOFIT and pedometer assessment, participants from the intervention group alone were observed at two time points (first and last yoga PE class). To ensure validity, pre-intervention observations were conducted by two research assistants (RAs) during the first yoga class of the semester.

### 2.2. Procedure

Before the yoga PE class, the yoga instructor introduced the research assistants (RAs) to the class and briefly explained the purpose of the study. RAs then securely attached pedometers onto students’ waistlines near the right hip. Prior to observation, four children (two males, two females) were randomly chosen. Observation began when over half of the class was present. Once instruction began, each RA listened to the SOFIT pacing recording and began their observation. Each student was observed during four-minute intervals. Observation ceased once the instructor announced completion of class. A binder was provided to RAs as a reference for accurate rating of yoga poses. At the end of class, pedometers were collected, and pedometer data were recorded. Same procedures were again carried out during the last class at the end of the semester. Yoga PE class observation lasted, between 20 and 30 min.

All students participating in the study, including intervention and control groups, were asked to complete a self-report questionnaire twice during the semester (pre-and post-intervention). The control group received self-report questionnaires at the same time points as the intervention group. Self-report assessments were only distributed to students in 4th, 5th, and 6th grade classes. Grades K-3 did not complete surveys due to potential difficulty in reading and understanding the survey’s content. Before distribution of the questionnaire, students were verbally told that if they felt uncomfortable participating in the survey, they could choose not to participate in the survey or skip any question that made them feel uncomfortable. Questionnaires were administered in class by RAs and took an average of 20 min to complete.

Time 1 (T1) represents baseline measurements taken Jan/Feb. The intervention group received surveys (grades 4–6), pedometers (grades K-6), and SOFIT (grades K-6). The control group only received surveys (grades 4–6). Time 2 (T2) represents post-test measurements taken April/May. The intervention group again received surveys (grades 4–6), pedometers (grades K-6), and SOFIT (grades K-6) while the control group only received surveys (grades 4–6). A total of 157 subjects were grouped by class into the intervention group (*n* = 10 classes) or control group (*n* = 2 classes), accordingly. PA levels and psychosocial variables were assessed through self-report surveys for intervention and control groups, but the intervention group was also observed via pedometers and SOFIT data. Therefore, there was no comparison group available to compare pedometer and SOFIT measurements obtained from the intervention group.

### 2.3. Intervention

#### 2.3.1. Intervention Group–Hatha Yoga

The intervention consisted of a Hatha yoga module lasting twelve weeks. The students received one yoga class lasting 20 to 30 min per week. During the yoga class, students were led through a modified Yoga Ed. lesson plan which included yoga breathing, poses, and relaxation techniques.

The yoga teacher had pre-existing yoga experience, was motivated to teach yoga to students, was certified by Yoga Ed. (now Breathe for Change) to teach the Yoga Ed. Children’s and Adolescent’s curriculum and used pre-made lesson plans made by Yoga Ed. for each developmental level. The Yoga Ed. curriculum meets National Physical Education Standards and supports student’s in developing physical, mental, emotional, cognitive, and social awareness and skills through yoga classes in the following ways:

Kindergarden: Supports key developmental milestones for young students, while promoting the development of students’ executive function and basic motor skills.

1st–3rd Grade: Plants the seeds for life skills and academic success, while cultivating important social and emotional skills.

4th–5th Grade: Plants the seeds for life skills and academic success, while developing resilience and executive function.

6th Grade: Supports the development of essential social skills while nurturing a positive self-image and equipping students with practical tools they can use both in and out of the classroom.

For more information please see: https://breatheforchange.com/about/curriculum/ (accessed on 3 June 2024).

#### 2.3.2. Control Group—Physical Education

The control group received regularly delivered physical education classes.

### 2.4. Measures

#### 2.4.1. Self-Report Questionnaires

Physical activity was measured using a modified version of the Godin Leisure-Time Physical Activity Questionnaire [12,13]. Participating students indicated how many days per week they engaged in strenuous, moderate, and mild PA for 0, 10, 20, 30, 40, 50, or 60+ minutes when they were not at school. Strenuous PAs were defined as activities that made the child’s heartbeat quicken and made them sweat. Examples of strenuous PAs were running, jogging, fast bicycling, and soccer. Moderate PAs were defined as activities that did not make participants tired but made them sweat. Examples of moderate PAs were fast walking, slow bicycling, easy swimming, and dancing. Mild PAs were defined as activities that required little effort and did not make participants sweat. Examples of mild PAs were easy walking, bowling, and fishing. Jacobs, Ainsworth, Hartman, and Leon [14], as well as Miller, Freedson, and Kline [11] reported excellent reliability and validity of the Leisure Time Physical Activity Questionnaire for adults. When compared to kilo calories expended per day in a sample of 5th, 8th, and 11th graders, Sallis, Buono, Roby, Micale, and Nelson [15] reported good test–retest reliability (r = 0.81) and adequate validity (r = 0.39). Additionally, several studies showed good ecological validity for the questionnaire in multiple different settings [16,17,18].

Sedentary behavior was determined by the number of hours participants watched television and played video games per day. Amount of screen time was measured by asking participants the following: “How many hours a day do you spend watching television and playing video games?” for 0, 1, 2, 3, 4, 5, 6, 7, 8, 9, 10+ hours per day. This measure which documents test–retest reliability [19] and validity has been shown to have a small negative correlation in PA with children [20].

For the assessment of psychosocial variables, the following items were adapted from measures used in Battista, Nigg, Chang, Yamashita, and Chung [21]. Participants indicated their attitude toward PA (i.e., “I believe I can be physically active every day” or “Physical activity is fun”) via a five-item Likert scale, answered on a scale of 1 = “disagree a lot” to 5 = “agree a lot”. Social Norm was measured by three items such as “Most people who are important to me think I should be physically active on a regular basis…” and scored on a five-point scale that ranged from (1) “disagree a lot” to (5) “agree a lot”. Self-efficacy was measured by responses to six questions such as “I feel confident that I can participate in regular physical activity when it is raining…” and scored on a five-point scale that ranged from (1) “not at all confident” to (5) “completely confident”.

Physical activity stage at baseline was measured using questions recommended by Nigg [22]. The specific responses were preceded by the definition of regular PA and the question, “Do you do regular physical activity as described above?” The response choices were “No, and I do not plan to start regular physical activity in the next six months” (pre-contemplation), “No, but I plan to start regular physical activity in the next 6 months” (contemplation), “No, but I plan to start regular physical activity in the next 30 days” (preparation), “Yes, I have been, but for less than six months” (action), and “Yes, I have been for more than six months” (maintenance). Students were instructed to select only one stage that best described their current PA pattern [21].

Perceived stress was determined by having students answer four questions pertaining to their feelings and thoughts during the last month. Participants answered questions such as “In the last month, how often have you felt that you were unable to control the important things in your life?” on a 5-point Likert-scale, ranging from 1 = “never” to 5 = “very often”. A study conducted by Roberti, Harrington, and Storch [23] found the perceived stress scale to be a reliable and valid instrument for college students.

#### 2.4.2. Pedometer

As a measurement tool to assess device-based PA, New Lifestyles Digi-Walker (model no. SW-701; NEW-LIFESTYLES, Inc. Lees Summit, MO, USA) pedometers were used to estimate steps per minute (SPM). Pedometers can be used with large numbers of individuals and help reduce the subjectivity in survey methods. Using direct observation as criterion measures, pedometers yield relatively high correlations (r = 0.80 to 0.97) [24]. Scruggs et al. [25] found that using SOFIT systematic observation as the criterion instrument and pedometers as the predictor instrument resulted in a high correlation (r = 0.74–0.86, *p* < 0.0001).

#### 2.4.3. System for Observing Fitness Instruction Time (SOFIT)

The SOFIT instrument was used to obtain simultaneous objective data on student activity levels and the lesson context in which the activity occurs during yoga classes (as the purpose did not address teacher behavior the SOFIT category of “instructor behavior” was not assessed). All SOFIT observers were trained to follow the SOFIT protocol by a certified SOFIT instructor. Student PA levels represent outcome variables. These include the number of minutes and percentage of lesson time spent in moderate-to-vigorous PA (MVPA). Lesson context was assessed as a process variable. Lesson context included minutes and percentage of lesson time spent in management, instruction, fitness, skill drills, game play, and other.

MVPA was coded as 1 = lying down; 2 = sitting; 3 = standing up; 4 = walking and 5 = vigorous. Vigorous indicated PA which corresponded to energy expenditure beyond what was needed for ordinary walking. The lesson context was assessed during a 10-s observation interval consisting of either general content (M) like transition, management, or a break; knowledge content (K) like physical fitness, general knowledge, rules, strategy, social behavior, or technique; or motor content comprising either fitness (F), skill practice (S), game play (G), or other (O) [25]. For each of the coded categories, sums were calculated at the end of the lesson and used for data analysis.

For data collection, pre-recorded verbal prompts on an MP3 player were used to keep observers on pace throughout a lesson via 10-s observe/record prompts. Student activity and lesson context were coded after the “record” prompt.

Five target students, consisting of two males, two females, and one back-up student, were selected randomly before the lesson started. Each student was observed for 4 consecutive minutes before the focus was changed to the next student. This procedure was repeated until the end of the lesson. SOFIT has been recommended for use in research and assessment of PA levels in PE classes [26]. In addition, SOFIT accurately differentiates between students’ sedentary behaviors and MVPA behaviors [27].

### 2.5. Data Analyses

Data entry and analysis were conducted using the Statistical Package for the Social Sciences (SPSS) data analysis program, version 20.0. In an attempt to minimize data entry errors, we implemented screening and cleaning techniques.

Repeated measures ANOVAs were utilized for data collected from self-report questionnaires. Descriptive statistics were calculated for pedometer data and a *t*-test was applied to test for statistically significant differences from Time 1 to Time 2. Regarding SOFIT, we tested statistical significance using the Chi-Square test with the *p* level set at 0.05.

## 3. Results

### 3.1. Self-Report Questionnaires

All but one of the self-reported indicators were in favor of the intervention group, although none showed statistically significant differences between the groups (all *p*-values > 0.05; see Table 2). Though the improvements were minimal, the intervention group reported greater increases in comparison to the control group for six of the seven indicators used in the survey, including MVPA, sedentary behavior, self-efficacy, social norm, attitude, and stress. The control group reported a greater increase for the intention indicator than the intervention group.

### 3.2. Pedometers

The pedometer data were collected for the intervention group only and therefore had no control group for comparison. The pedometer results indicated an increase in the number of steps per minute during the yoga classes (Time 1 = 6.43 steps/min, SD = 3.75; Time 2 = 7.29 steps/min, SD = 3.82; t = 1.84, *p* = 0.068, d = 0.273).

### 3.3. SOFIT

SOFIT data were collected for the intervention group only and therefore there was no control group for comparison. Baseline analyses of the SOFIT data indicated that most of the class time (38.10%) was spent on knowledge (Table 3). This was followed by fitness and skill practice which both took up 28.40% of the total class time. When comparing Time 1 and Time 2, the largest decrease in class time spent was in the category of fitness. Fitness is defined in terms of individual cardiovascular endurance, strength, or flexibility. The amount of time dedicated to knowledge also decreased while the greatest increase observed was for skill practice. Knowledge is defined as students participating in the acquisition of new knowledge as opposed to engaging in activity. Skill practice refers to developing a certain skill where time is allotted for instruction and feedback. However, fitness requires less instruction and is more focused on PA engagement.

The PA portion of the SOFIT results revealed a significant difference for activity from the baseline at Time 1 to the follow-up at Time 2 (X^2^(16) = 36.14, *p* = 0.003, φ = 0.480). The major differences were a decrease in sitting and very active behavior and an increase in walking (all differences >5%; see Table 4).

## 4. Discussion

There are few studies regarding the evaluation of a yoga intervention program [28] that specifically follows the state mandated physical education standards. Our aim in this study was to determine if participation in yoga influenced PA and improved psychosocial variables. Mendelson et al. [29] suggest that mindfulness-based approaches to intervention programs may be beneficial for youths by enhancing their responses to stress. Breathing lies at the heart of yoga so many breathing exercises and techniques are utilized in yoga, which are fundamental to meditation. Given this relationship between yoga and meditation, Black, Milam, and Sussman [30] concluded that this type of concentration of the mind, when used as treatment for physiological, psychosocial, and behavioral conditions among youths, is an effective form of intervention. Similarly, Malar and Maniazhagu [31] showed that integrative neuromuscular training combined with yoga improved abdominal strength endurance in primary school children. Further, teaching youth yoga techniques, mindfulness, and emotion regulation improved emotional regulation and academic outcomes [32]. A yoga lesson per week over eight weeks program also improved emotional regulation and decreased aggression in elementary school children [33]. Extending the findings to a comparison physical education, our self-reported survey data showed that a Hatha yoga program was at least as good as a PE program, although it is important to note that our control group did not include pedometer and SOFIT data.

The results regarding the pedometer data indicated a trend towards statistical significance and the self-report survey data were not statistically significant, which we attribute to our small sample size. Although we found no significant differences in the self-report survey results, it is worth considering that the majority of these measurements including self-efficacy, intention to be physically active, social norm, attitude toward PA began (baseline) and remained (follow-up) at high values. For example, attitude toward PA remained high and unchanged over time for the intervention group (4.66 on a 5-point Likert scale), which may reflect a ceiling effect and thus is difficult to increase.

At the beginning of this yoga program, students were first introduced to yoga postures and techniques through verbal instruction and physical demonstration meaning the students were not very familiar with yoga; rather, they were taking part in the knowledge portion of learning yoga techniques. As the program progressed so did the ability of each student to perform yoga postures and techniques correctly and successfully. This is evident in our results as the amount of class time spent decreased for knowledge and increased for skill practice from Time 1 to Time 2. In a study conducted on high school students, investigators found game play to be the leading context (47%), and knowledge and skill development received little time commitment [26,34].

Overall, our results suggest that there was an increasing trend in PA among students participating in this yoga intervention program. To address our aim of stress reduction, however, there was no significant evidence to support this hypothesis. It needs to be considered that this evidence was self-reported by the students, had we been capable of measuring stress using physiological measures like cortisol swabs our results may have turned out to be more accurate regarding our aim of lowering stress levels. Within the emerging yoga research field, it is imperative to consider other and perhaps complementary determinants of stress reduction. Ideally, future research should examine various determinants of stress reduction to gain a better understanding of which determinants are affected by the practice of yoga. Finally, we found little to no difference between the intervention and control groups; therefore, within our assessed outcomes, yoga as a form of physical education is no worse or just as good as standard physical education in delivering an acceptable PA program.

### Limitations

Our most challenging difficulty was obtaining parental consent. Considering that we gave the consent forms to classroom teachers to be handed out to students, the percentage of returned consent forms (56%) was relatively low, which could be due to a number of factors, and suboptimal consent has generalizability implications. Nonetheless, communication between yoga instructors, administration, and classroom teachers is vital for the success of the intervention. Also, social desirability bias needs to be considered for the self-report survey. Another significant limitation was the fact that the intervention group did not have a comparison group for the measurements using the pedometers and SOFIT due to a lack of resources. We would have liked to include all grade levels in the control group and observe them in their regular physical education environment in comparison to their yoga counterparts, though it was not feasible for this particular study.

Utilizing pedometers as a measure of PA allowed us to find an increasing trend in the students’ number of steps taken per minute; however, since yoga uses a multitude of movements not limited to step motion of the hips, we may have benefited by additionally utilizing an accelerometer in order to account for the many other movements that yoga contributes to PA. Although it was not feasible for this particular study, there is a time-stamped technology within accelerometers that present movement as a rate, which allows researchers to infer the amount of time spent at various levels of PA intensity [35].

## 5. Conclusions

In today’s age of technology and information it is not hard to imagine children as having a difficult time trying to focus on particular endeavors, given their infinite number of distractions [36,37]. Mindfulness-based programs such as this yoga intervention focus on cultivating the sharpness of the mind and attempt to strengthen the physical body and spirit [7,9,38]. Yoga caters to balance, strength, and flexibility through focusing on the pliability of the body and mind, which is practiced through postures, breath, and meditation [38].

Given our observation data while considering the limitations in our study design, the results of this study did not demonstrate a significant difference between the intervention and control groups in terms of increasing PA levels or improving psychosocial variables. Future studies should use these findings to improve and advance research tools related to the health effects of yoga as a form of physical education among school-age children. Perhaps researchers should examine various methods of measuring mindfulness and the effect that yoga may have on the human psyche. Regarding PA, investigators can consider the six areas of fitness, which could contribute to more concrete evidence, rather than simply measuring students’ levels of PA. Furthermore, this field of research should investigate the potential benefits of integrating yoga and standard physical education as a combination class.

The results of this study may be appropriate for school-aged children and schools that find difficulty in dealing with inattentive/inactive students. There are currently around 940 schools in the United States that are implementing yoga in the classroom, and around 5400 yoga instructors have been trained to provide yoga in educational settings, meaning that yoga may be increasingly accessible to study in elementary schools and there are increasing objective and subjective data on the topic [39]. As there is growing research on the effects of yoga on school-aged children, yoga may prove to be an excellent vessel for students struggling with low levels of PA and poor psychosocial perceptions.

## Figures and Tables

**Table 1:** Group assignment for yoga (intervention) and physical education (control) program evaluation.

Grade (# of Classes)	Intervention Group # of Classes	Control Group # of Classes
K (1)	1	-
1 (3)	3	-
2 (1)	1	-
3 (1)	1	-
4 (2)	1	1
5 (2)	2	-
6 (2)	1	1
**Total**	**10**	**2**

Note: K—kindergarden.

**Table 2:** Self-reported indicators acquired from survey instrument comparing Time 1 and Time 2.

Variable	Group	Time1		Time 2		Repeated Measures ANOVA		
		Mean	St. Dev	Mean	St. Dev	F (df)	*p* Value	η^2^
MVPA	Control	5.64	2.56	6.16	2.92	0.14 (1, 54)	0.71	
	Intervention	5.43	2.84	6.30	2.67			0.003
Sedentary Behavior	Control	1.62	1.32	1.77	1.58	0.14 (1, 58)	0.71	
	Intervention	2.11	1.52	2.13	1.67			0.002
Self-efficacy	Control	4.62	0.77	4.80	0.44	0.85 (1, 59)	0.36	
	Intervention	4.41	0.86	4.77	0.47			0.014
Intention	Control	4.23	1.23	4.61	0.65	0.22 (1, 59)	0.64	
	Intervention	4.43	0.89	4.68	0.62			0.004
Social Norm	Control	4.08	1.32	3.92	1.26	0.54 (1, 59)	0.46	
	Intervention	4.02	1.25	4.06	1.01			0.009
Attitude	Control	4.76	0.44	4.77	0.59	0.01 (1, 59)	0.92	
	Intervention	4.64	0.70	4.67	0.55			0.000
Stress	Control	3.59	0.70	3.73	0.70	0.17 (1, 58)	0.68	
	Intervention	3.37	0.78	3.42	0.65			0.003

Note: In the overall model, Time is Statistically Significant (*p* = 0.000). MVPA = moderate to vigorous physical activity.

**Table 3:** Curricular lesson context for yoga physical education intervention program using SOFIT *, showing the relationship from Time 1 to Time 2.

Time 2								
			Management	Knowledge	Fitness	Skills	Other	Total
**Time 1**	management	count	3	8	1	23	1	36
		% of Total	0.40%	1.20%	0.10%	3.30%	0.10%	5.20%
	knowledge	count	18	81	38	122	4	263
		% of Total	2.60%	11.70%	5.50%	17.70%	0.60%	38.10%
	fitness	count	14	66	9	95	12	196
		% of Total	2.00%	9.60%	1.30%	13.70%	1.70%	28.40%
	skills	count	17	52	22	100	5	196
		% of Total	2.50%	7.50%	3.20%	14.50%	0.70%	28.40%
**Total**		**count**	**52**	**207**	**70**	**340**	**22**	**691**
		**% of Total**	**7.50%**	**30.00%**	**10.10%**	**49.20%**	**3.20%**	**100.00%**

* SOFIT—System for Observing Fitness Instruction Time. X^2^(12) = 25.98; *p* = 0.011.

**Table 4:** Count and percentage of students participating at various levels of physical activity.

Variable		Time 1	Time 2
lying	Count	97	106
	% of Total	14.00%	15.30%
sitting	Count	228	186
	% of Total	32.90%	26.80%
standing	Count	228	186
	% of Total	16.90%	20.00%
walking	Count	111	169
	% of Total	16.00%	24.40%
very active	Count	141	94
	% of Total	20.30%	13.50%
Total	Count	694	694
	% of Total	100.00%	100.00%

X^2^(16) = 36.14; *p* = 0.003.

## Data Availability

Data is contained within the article.

## References

[1] Sallis J.F., McKenzie T.L., Kolody B., Lewis M., Marshall S., Rosengard P. (1999). Effects of health-related physical education on academic achievement: Project SPARK. Res. Q. Exerc. Sport.

[2] Kalish L., Guber T. (2001). Yoga Ed K-8 Yoga Program and Curriculum.

[3] Hagins M., Haden S.C., Daly L.A. (2013). A randomized controlled trial on the effects of yoga on stress reactivity in 6th grade students. Evid.-Based Complement. Altern. Med..

[4] Chaya M.S., Nagendra H., Selvam S., Kurpad A., Srinivasan K. (2012). Effect of yoga on cognitive abilities in schoolchildren from a socioeconomically disadvantaged background: A randomized controlled study. J. Altern. Complement. Med..

[5] Ferreira-Vorkapic C., Feitoza J.M., Marchioro M., Simões J., Kozasa E., Telles S. (2015). Are there benefits from teaching yoga at schools? A systematic review of randomized control trials of yoga-based interventions. Evid.-Based Complement. Altern. Med..

[6] Innes K.E., Selfe T.K. (2016). Yoga for adults with type 2 diabetes: A systematic review of controlled trials. J. Diabetes Res..

[7] Granath J., Ingvarsson S., von Thiele U., Lundberg U. (2006). Stress management: A randomized study of cognitive behavioural therapy and yoga. Cogn. Behav. Ther..

[8] Aljasir B., Bryson M., Al-Shehri B. (2010). Yoga practice for the management of type II diabetes mellitus in adults: A systematic review. Evid.-Based Complement. Altern. Med..

[9] Pilkington K., Kirkwood G., Rampes H., Richardson J. (2005). Yoga for depression: The research evidence. J. Affect. Disord..

[10] Cramer H., Lauche R., Langhorst J., Dobos G. (2013). Yoga for depression: A systematic review and meta-analysis. Depress. Anxiety.

[11] Miller D.J., Freedson P.S., Kline G.M. (1994). Comparison of activity levels using the Caltrac accelerometer and five questionnaires. Med. Sci. Sports Exerc..

[12] Godin G., Shephard R.J. (1985). A simple method to assess exercise behavior in the community. Can. J. Appl. Sport Sci..

[13] Godin G.A., Jobin J.E., Bouillon J. (1986). Assessment of leisure time exercise behavior by self-report: A concurrent validity study. Can. J. Public Health = Rev. Can. De Sante Publique.

[14] Jacobs D.R., Ainsworth B.E., Hartman T.J., Leon A.S. (1993). A simultaneous evaluation of 10 commonly used physical activity questionnaires. Med. Sci. Sports Exerc..

[15] Sallis J.F., Buono M.J., Roby J.J., Micale F.G., Nelson J.A. (1993). Seven-day recall and other physical activity self-reports in children and adolescents. Med. Sci. Sports Exerc..

[16] Amireault S., Godin G. (2015). The Godin-Shephard leisure-time physical activity questionnaire: Validity evidence supporting its use for classifying healthy adults into active and insufficiently active categories. Percept. Mot. Ski..

[17] Amireault S., Godin G., Lacombe J., Sabiston C.M. (2015). Validation of the Godin-Shephard Leisure-Time Physical Activity Questionnaire classification coding system using accelerometer assessment among breast cancer survivors. J. Cancer Surviv..

[18] Amireault S., Godin G., Lacombe J., Sabiston C.M. (2015). The use of the Godin-Shephard Leisure-Time Physical Activity Questionnaire in oncology research: A systematic review. BMC Med. Res. Methodol..

[19] Buckworth J., Nigg C. (2004). Physical activity, exercise, and sedentary behavior in college students. J. Am. Coll. Health.

[20] Nigg C.R. (2005). There is more to stages of exercise than just exercise. Exerc. Sport Sci. Rev..

[21] Battista J., Nigg C.R., Chang J.A., Yamashita M., Chung R. (2005). Elementary after school programs. Californian J. Health Promot..

[22] Nigg C.R., Welk G.J. (2002). Physical activity assessment issues in population based interventions: A stage approach. Physical Activity Assessments for Health-Related Research.

[23] Roberti J.W., Harrington L.N., Storch E.A. (2006). Further psychometric support for the 10-item version of the perceived stress scale. J. Coll. Couns..

[24] Sirard J.R., Pate R.R. (2001). Physical activity assessment in children and adolescents. Sports Med..

[25] Scruggs P.W., Beveridge S.K., Eisenman P.A., Watson D.L., Shultz B.B., Ransdell L.B. (2003). Quantifying physical activity via pedometry in elementary physical education. Med. Sci. Sports Exerc..

[26] McKenzie T. (2009). SOFIT Generic Description and Procedures Manual. https://www.researchgate.net/publication/318276946_SOFIT_System_for_Observing_Fitness_Instruction_Time_Description_and_Procedures_Manual_Generic_Version.

[27] Rowe P., van Der Mars H., Schuldheisz J., Fox S. (2004). Measuring students’ physical activity levels: Validating SOFIT for use with high-school students. J. Teach. Phys. Educ..

[28] Agudo Villarejo C.A., Zagalaz J.C., Navío E.P., Sánchez A.J.L. (2024). Beneficios del yoga para la educación postural: Propuesta de programa de intervención para educación primaria. (Benefits of yoga for postural education: Proposed intervention program for elementary education). Retos.

[29] Mendelson T., Greenberg M.T., Dariotis J.K., Gould L.F., Rhoades B.L., Leaf P.J. (2010). Feasibility and preliminary outcomes of a school-based mindfulness intervention for urban youth. J. Abnorm. Child Psychol..

[30] Black D.S., Milam J., Sussman S. (2009). Sitting-meditation interventions among youth: A review of treatment efficacy. Pediatrics.

[31] Malar S., Maniazhagu D. (2020). Effects of Integrative Neuromuscular Training Combined with Yoga and Stretching Exercises on Abdominal Strength Endurance of Primary School Children. Indian J. Public Health Res. Dev..

[32] McCurdy B.H., Bradley T., Matlow R., Rettger J.P., Espil F.M., Weems C.F., Carrion V.G. (2024). Program evaluation of a school-based mental health and wellness curriculum featuring yoga and mindfulness. PLoS ONE.

[33] Kafi H., Tavakoli V.M. (2021). The Effectiveness of Yoga Therapy on Emotional Regulation and Aggression in Elementary School Children. Iran. J. Psychiatr. Nurs..

[34] Smith N.J., Lounsbery M.A., McKenzie T.L. (2014). Physical activity in high school physical education: Impact of lesson context and class gender composition. J. Phys. Act. Health.

[35] Adams M.A., Johnson W.D., Tudor-Locke C. (2013). Steps/day translation of the moderate-to-vigorous physical activity guideline for children and adolescents. Int. J. Behav. Nutr. Phys. Act..

[36] Harrison J.R., Vannest K., Davis J., Reynolds C. (2012). Common problem behaviors of children and adolescents in general education classrooms in the United States. J. Emot. Behav. Disord..

[37] Miller R. (2012). The Double D’s of Destruction: How Our Distracted and Desensitized Consciousness Is Destroying Our Communities and Failing Our Children.

[38] McCall M.C. (2013). How might yoga work? An overview of potential underlying mechanisms. J. Yoga Phys. Ther..

[39] Butzer B., Ebert M., Telles S., Khalsa S.B. (2015). School-based yoga programs in the United States: A survey. Adv. Mind-Body Med..

